# Qualifying as an Academic Child and Adolescent Psychiatrist During a Pandemic: Top Tips for Trainee Led Training and Research Activities

**DOI:** 10.1192/j.eurpsy.2022.195

**Published:** 2022-09-01

**Authors:** A. Seker

**Affiliations:** Cambridge and Peterborough NHS Foundation Trust, Child/adolescent Psychiatry, Cambridge, United Kingdom

## Abstract

Combining clinical and academic roles during psychiatric training can be challenging but equally enriching. However, the Covid-19 pandemic has complicated many aspects of psychiatric training, including changes of service provision, clinical duties, training activities and academic opportunities. In this interactive Workshop session, tips and strategies for balancing academic and clinical responsibilities during psychiatric training will be discussed based on real-life experiences and literature recommendations. The impact of the pandemic and what skills could be useful to flexibly tackle the challenges that come with an ongoing public health crisis while working to satisfy academic and clinical requirements in a set period of time will also be considered. The speaker will also include additional resources to improve such soft skills for participants.

**Disclosure:**

No significant relationships.

